# Clinical profile and outcomes of young women with denovo-metastatic breast cancer: real-world data from a tertiary care centre in India

**DOI:** 10.3332/ecancer.2025.1932

**Published:** 2025-06-24

**Authors:** Sushmita Rath, Mehak Trikha, Laboni Sarkar, Kunal Jobanputra, Akash Pawar, Revathy Krishnamurthy, Ayushi Sahay, Ayushi Sahay, Purvi Thakkar, Sneha Shah, Venkatesh Kapu, Anbarasan Sekar, Prabhat Bhargava, Seema Gulia, Rima Pathak, Tabassum Wadasadawala, Rajiv Sarin, Rajendra Badwe, Sudeep Gupta, Jyoti Bajpai

**Affiliations:** 1 Department of Medical Oncology, Tata Memorial Centre, Homi Bhabha National Institute (HBNI), Mumbai, India; 2 Department of Biostatistics, Tata Memorial Centre, Homi Bhabha National Institute (HBNI), Mumbai, India; 3 Department of Radiation Oncology, Tata Memorial Centre, Homi Bhabha National Institute (HBNI), Mumbai, India; 4 Department of Pathology, Tata Memorial Centre, Homi Bhabha National Institute (HBNI), Mumbai, India; 5 Department of Surgical Oncology, Tata Memorial Centre, Homi Bhabha National Institute (HBNI), Mumbai, India; 6 Department of Nuclear Medicine, Tata Memorial Centre, Homi Bhabha National Institute (HBNI), Mumbai, India; 7 Department of Radiodiagnosis, Tata Memorial Centre, Homi Bhabha National Institute (HBNI), Mumbai, India; † Authors contributed equally to the work.

**Keywords:** denovo metastatic breast cancer, young breast cancer, survival outcomes, real world data

## Abstract

**Background:**

Denovo metastatic young breast cancer (dnmYBC), defined as age <40 years, is a challenging entity, with a significant burden and sparse data from low and middle-income countries.

**Method:**

We analysed the prospectively collected data of dnmYBC women from 2015 to 2016.

**Results:**

There were 188 dnmYBC with a median age of 35.5 years. Of these, hormone receptor positive (HR+) were 72 (38.3) %, triple-negatives (TNBC) were 45 (23.9) %, Human Epidermal Growth Factor Positive (HER2+) were 42 (22.4) % and triple positives were 29 (15.4) %. TNBC women predominantly had visceral 40 (88.9%) metastasis, HR+ had nodal 51 (70.8%) and skeletal 10 (13.8%), while HER2+ women had higher brain metastasis (BM) 16 (38.1%).

At a median follow-up of 39.8 [Interquartile range (IQR): 24–55.5] months, the median event-free survival (EFS) was 9.3 (95% CI; 8.1–10.4) months for the entire cohort and 1-year, 2-year and 3-year predicted EFS were 47.8%, 13.4% and 3%, respectively. The median EFS was superior in HR+ women.

[15.7 months, hormone receptor (HR)−0.53;95% CI-9.8–21.7; p-0.013] versus (11.4 months, 95 %CI-5.9–16.8) in TNBC versus (7.7 months, 95% CI-6.0–9.5) in HER-2 + women and without BM at baseline [9.3 versus 3.0 months (with BM), HR-5.65; CI-1.72–17.9; *p*-0.001]. Median EFS was superior in the treatment-naïve (155, 82.4%) versus prior-treated (33, 17.5%) women, 35.5 (95% CI:12.24–58.72) versus 12.4 (95% CI:11.45–13.51) months; *p*-0.000]. The HER2+ women who received targeted therapy in the first line had a significantly superior median EFS of 13.0 versus 7.7 months (HR -0.465:CI 0.22–0.57: *p*-0.038).

**Conclusion:**

Denovo mYBC is associated with an aggressive course, poor prognosticators include HR negative disease, brain metastasis, inadvertent prior treatment and inadequate access to targeted therapies. Early diagnosis, prompt treatment and expanding accessibility are warranted to improve care.

## Introduction

Breast cancer is the most common malignancy among women both globally and in India, accounting for 13.5% of all new cancer cases and 10.6% of all cancer-related deaths, with a cumulative risk of 2.81 as per GLOBOCAN 2020 [1]. In India, there is a growing trend towards a younger age at diagnosis compared to the Western population [2, 3]. Women diagnosed with breast cancer at ≤40 years and ≤35 years of age are defined as young breast cancer (YBC) and very-YBC (v-YBC), respectively [4–6]. While early-stage YBC has a favourable 5-year overall survival (OS) exceeding 90% for stage I and II, the prognosis for metastatic disease remains dismal, with a 5-year OS of only 24% in stage IV [7]. The survival disparity in lower-middle-income countries (LMICs) is further accentuated by a higher proportion of higher proportion of advanced-stage presentation, delayed diagnosis and suboptimal access to targeted therapies [8]. Additionally, tumours in younger women exhibit have poor prognostic features, including higher hormone receptor (HR) negativity, varied biological and aggressive molecular profiles, leading to higher mortality rates [9–14]. Denovo Metastatic breast cancer (dnMBC) is defined as the presence of distant metastasis at initial diagnosis. It is clinically and biologically distinct from recurrent metastatic breast cancer [18]. In high-income countries (HIC), dnMBC accounts for 6%–10% of new breast cancer diagnoses [18–21]. However, the incidence is higher in LMICs, potentially due to delayed presentation, healthcare access barriers and cultural or logistical factors influencing health-seeking behaviours [8, 22]. The dnMBC phenotype is associated with younger age at diagnosis, higher prevalence of visceral and brain metastases and often displays aggressive tumour biology [13, 14, 19–21]. Despite its clinical importance, dnMBC remains an understudied entity in the global literature, particularly in the LMIC context. Hence, we present the largest single-centre analysis from India focused on denovo metastatic YBC (dnmYBC). This study aims to evaluate the clinicopathologic features, treatment patterns and survival outcomes in this understudied population, thereby contributing novel insights with implications for LMICs and similar healthcare settings.

## Material and methods

This was a retrospective, single-centre study conducted at a tertiary Centre in India. The study population included women ≤40 years of age with histologically diagnosed with dnMBC, and had received at least partial treatment at our institute between January 1, 2015, and December 31, 2016. Patients with oligometastatic disease (defined as limited metastatic burden treated with curative intent) who were previously reported in an earlier analysis [17] were excluded from this study to maintain cohort uniformity and avoid overlap with prior findings.

### Data collection and variables

Demographic details, clinical parameters, tumour characteristics and treatment data were retrieved from the electronic medical records and supplemented with telephonic follow-up when needed. Tumour staging was done as per the American Joint Committee on Cancer 7th edition TNM classification [43].

Tumour subtypes were characterised based on immunohistochemistry testing of estrogen receptor (ER), progesterone receptor (PR) and human epidermal growth factor receptor 2 (HER2) expression. Hormone receptor positive (HR+) status was defined as % ≥1 of cell staining for ER or PR [15]. HER2 positivity was determined according to the College of American Pathologists guidelines [16]. Tumours negative for ER, PR and HER2 were classified as triple negative breast cancer (TNBC), while tumours positive for ER, PR and HER2 were classified as triple positive (TP) breast cancer. Response to therapy was assessed using clinical and radiological evaluation, and not strictly based on response evaluation criteria in solid tumours (RECISTs) criteria due to real-world variability. The objective response rate (ORR) was defined as the proportion of patients achieving a complete or partial response to treatment based on RECIST version 1.1 [44]. Chemotherapy-related adverse events were graded in accordance with the National Cancer Institute’s Common Terminology Criteria for Adverse Events, version 3.0 [45]. Chemotherapy-related toxicity was analysed only for patients who received systemic chemotherapy at our institution and had complete adverse event documentation available in the medical records. Women who received partial treatment elsewhere or those managed without chemotherapy (Endocrine therapy alone) were excluded from the toxicity assessment due to incomplete adverse reporting.

### Statistical analysis

All statistical analysis were performed using International Business Machine (IBM) SPSS Software version 25 IBM and R studio version 2023.03.0. Descriptive statistics for demographic, tumour and treatment-related characteristics were presented in frequencies and percentages. Event-free survival (EFS) was defined as the duration from the date of registration to the date of disease progression. OS was defined as the time from the date of registration to the date of death from any cause or the date of the last follow-up. Patients lost to follow-up were appropriately censored at their respective last follow-up dates. Survival curves were generated using Kaplan–Meier method. Median survival estimates and survival rates at 1, 2, 3 and 5 years were calculated along with 95% confidence interval (CI), using Greenwood’s formula. The log-rank test was used to compare the survival between the two independent groups. Specifically, survival outcomes were compared across the following variables: receptor subtypes [HR+, Human Epidermal Growth Factor Positive (HER2+), TNBC and triple-positive], presence or absence of brain metastasis at baseline, treatment-naïve versus prior-treated status and receipt of anti-HER2 targeted therapy in the first-line versus subsequent lines of treatment.

Univariate cox proportional hazards regression model was applied to estimate hazard ratio and corresponding 95% CIs for factors associated with the OS and EFS. Statistical significance was determined using a two-sided *p* value of less than 0.05.

## Context summary

### Key objective

The current study aims to explore the demographics, treatment patterns, prognosis and survival outcomes of young women with dnMBC in India.

### Knowledge generated

Denovo mYBC is associated with an aggressive course, poor prognosticators include HR negative disease, brain metastasis, inadvertent prior treatment and inadequate access to targeted therapies.

### Relevance of the study

Largest single-centre study in denovo-metastatic YBC. First study from India with denovo metastatic YBC with results that are widely applicable, inclusive of other LMICs.

## Results

### Host and tumour characteristics

**Figure 1.**
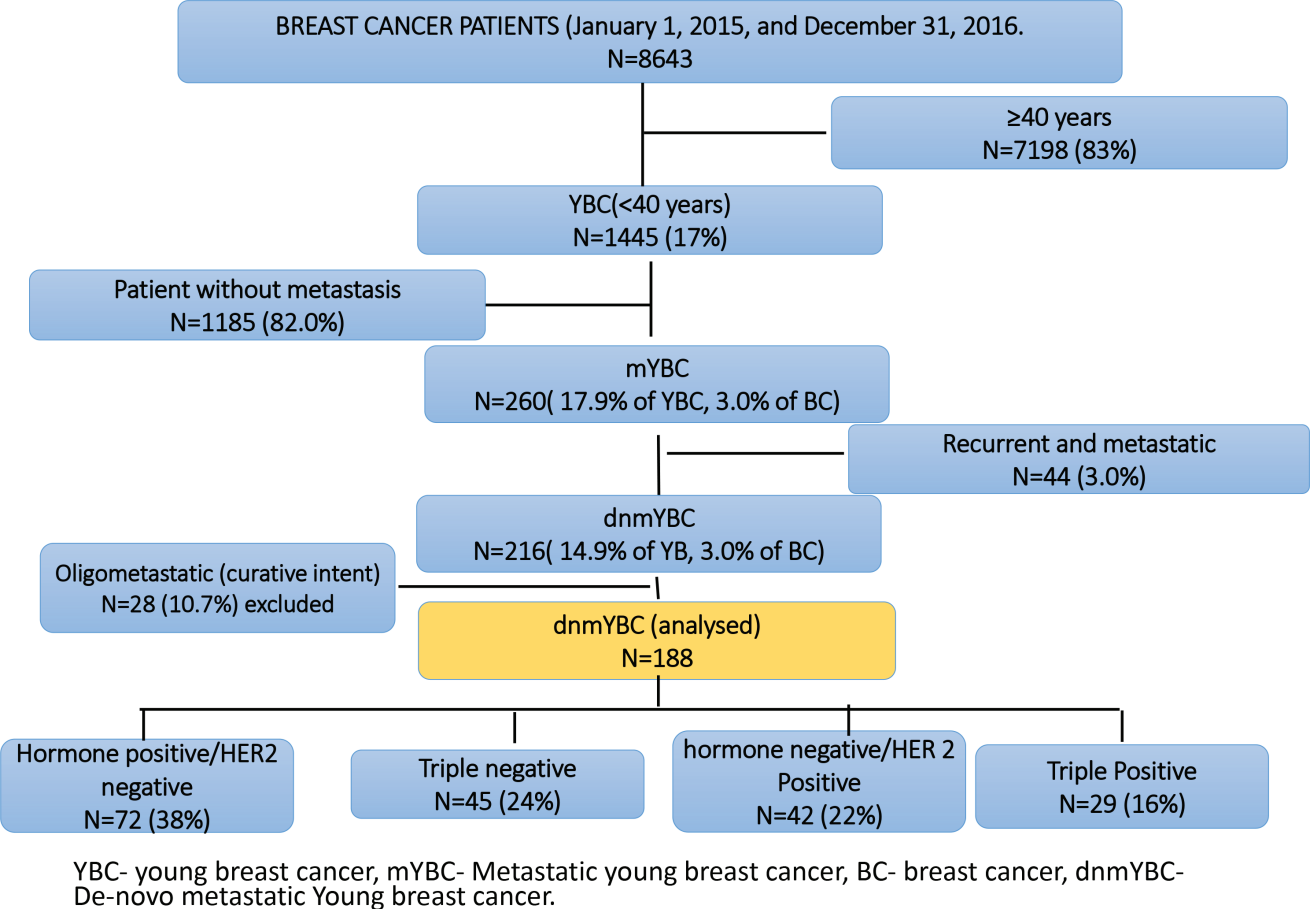
Consort diagram.

Of the 8634 women with breast cancer registered at our centre during the study period, 1445 (16.7%) were YBC. Among them, 1185 (82.0%) were non-metastatic and 260 (18.0%) were metastatic, inclusive of 44 (3.0%) who were recurrent metastatic and 216 (15.0%) who were dnMBC. Out of 216 patients, 28 (10.7%) women with oligometastatic dnmYBC were treated with curative intent and were reported earlier and excluded from the current analysis [17]. Hence, the current analysis included 188 women with dnmYBC registered during the study period (Figure 1).

**Figure 2.**
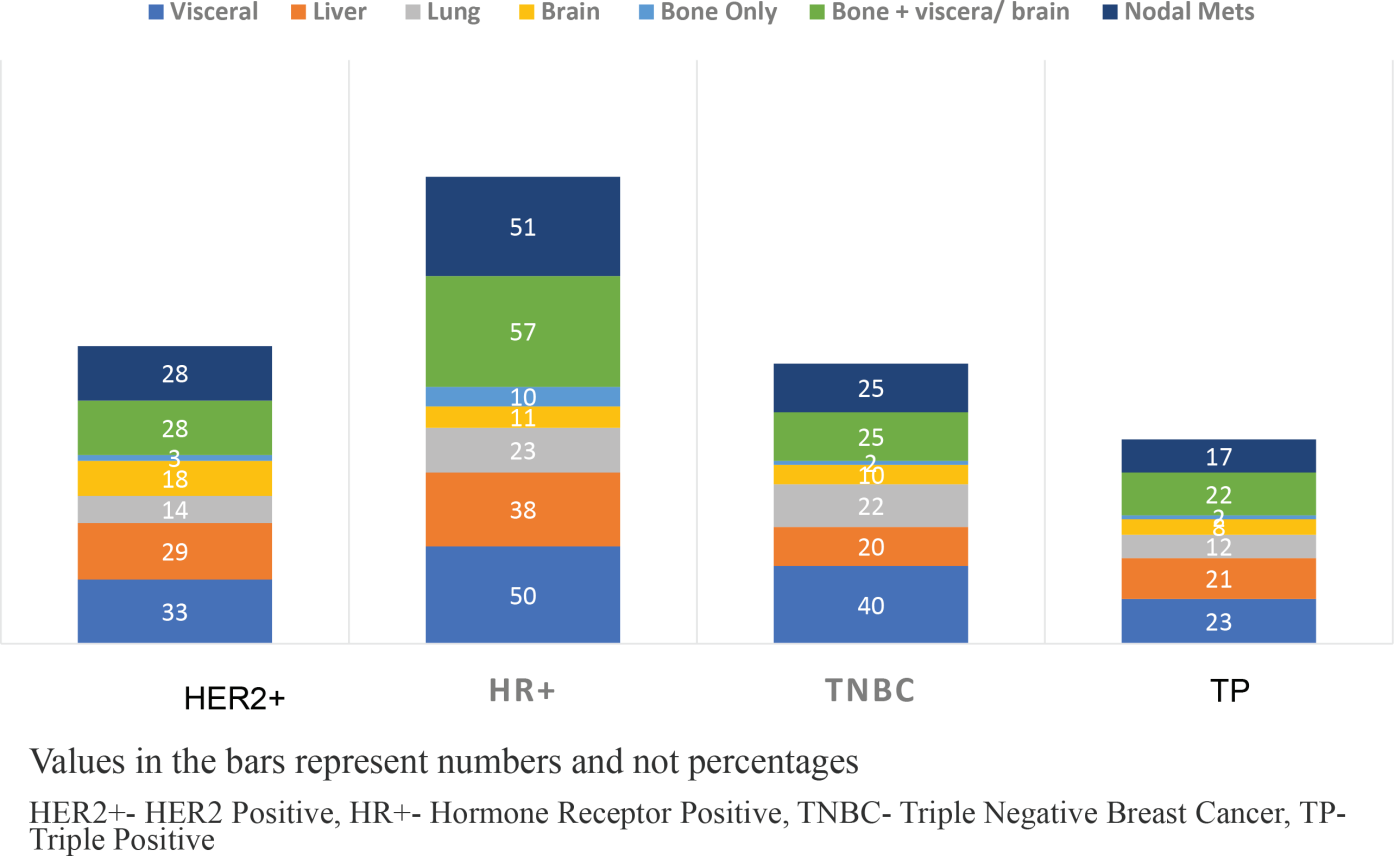
Incidence of metastasis according to hormone receptor status.

The median age of the cohort was 35.5 IQR:31–38) years. The tumour subtypes included HR+ in 72 (38.3%), TNBC in 45 (23.9%), HER2+ in 42 (22.4%) and TP in 29 (15.4%) women. 177 (91.1%) of the women presented with large tumour sizes (≥5 cm). Solitary skeletal metastasis was observed in 17 (9.0%) women and visceral metastasis was seen in 146 (77.0%). The commonest sites of visceral metastasis, were liver in 108 (57.4%), lung in 71 (37.7%), nodal metastasis in 67 (35.5%) and brain in 4 (2.1%) patients. Since some patients had multiple sites of metastasis, the total number does not add upto 188. The distribution of the sites of metastasis is given in Table 1. The receptor subtypes with the highest propensity to develop brain metastasis was HER2+ in 16 (38.1%) patients, for visceral metastasis, it was TNBC subtype in 40 (88.9%) and for skeletal-10 (13.8%) and nodal 51 (70.8%) metastasis, it was HR+ subtype. Among the cohort, 155 (82.4%) women were treatment-naïve, while 33 (17.5%) had received prior treatment at peripheral centres before presenting to our centre (Table 1, Figure 2).

## Treatment characteristics

### Hormone therapy

In terms of endocrine treatment, among the HR + cohort of 72 patients, 30 (41.7%) received single-agent hormone therapy in the first line, 28 (52.8%) received it in the second line and 14 (58.4%) patients received it in the third line, respectively. Additionally,13 (18.0%) women received ovarian suppression with leuprolide and 11 (15.3%) underwent radiotherapy ovarian ablation (RTOA).

### Chemotherapy

In terms of chemotherapy treatment, anthracyclines were given in 113 (60.1%) in first line,16 (8.5%) in second line and 16 (8.5%) in third line. Taxane-based chemotherapy was given to 32 (17.0%) patients in first-line, 49 (26.0%) in second line and 13 (6.9%) in third line. Forty-two (58.3%) women with HR+ MBC, received chemotherapy in the first-line setting, of which 12 (28.5%) women were in visceral crisis, 16 (38.0%) in impending visceral crisis and 9 (21.4%) started treatment from peripheral centres prior to presenting to our centre. The median number of therapy lines received was three (range 1–6). The detailed treatment characteristics are shown in Table 1 in the supplementary appendix.

### Targeted therapy

Ant-HER2 therapy was given to 13 (31.0%) women in first line, 21 (50.0%) in second line, 13 (31.0%) in third line and 9 (21.4%) women in fourth line. TDM1 was given to one patient in the fourth line. Ten (2.5%) women in the HR+ cohort received CDK4/6 inhibitors in the second and subsequent lines (Table S1).

The ORR, in terms of PR/SD/CR to the first-line, second-line, third-line, fourth line and fifth-line therapies in the entire cohort was 125/188 (66.4%), 87/90 (51.1%), 21/57 (36.8%), 8/28 (28.6%) and 4/14 (28.6%), respectively. Palliative radiotherapy to the bones was administered to 50/139 (33.5%) women. Of the 43 patients who developed brain metastases either at baseline or during follow-up, 30 (69.7%) received whole-brain radiotherapy.

### Survival analysis and factors affecting survival

At a median follow-up of 39.8 (95% CI = 24–55.5) months, 121 events were reported, inclusive of deaths, wherein one was attributed to chemotherapy toxicity. The Median EFS was 9.3 (95% CI = 8.1–10.4) months. The median EFS was superior in HR+ women (15.7 months, HR-0.53;95% CI-9.8–21.7; *p*-0.013) versus (11.4 months, 95 %CI-5.9–16.8) in TNBC versus (7.7 months, 95% CI-6.0–9.5) in HER-2 + women and without brain metastasis at baseline [9.3 versus 3.0 months, HR-5.65; CI-1.72–17.9; *p*-0.001].

**Figure 3.**
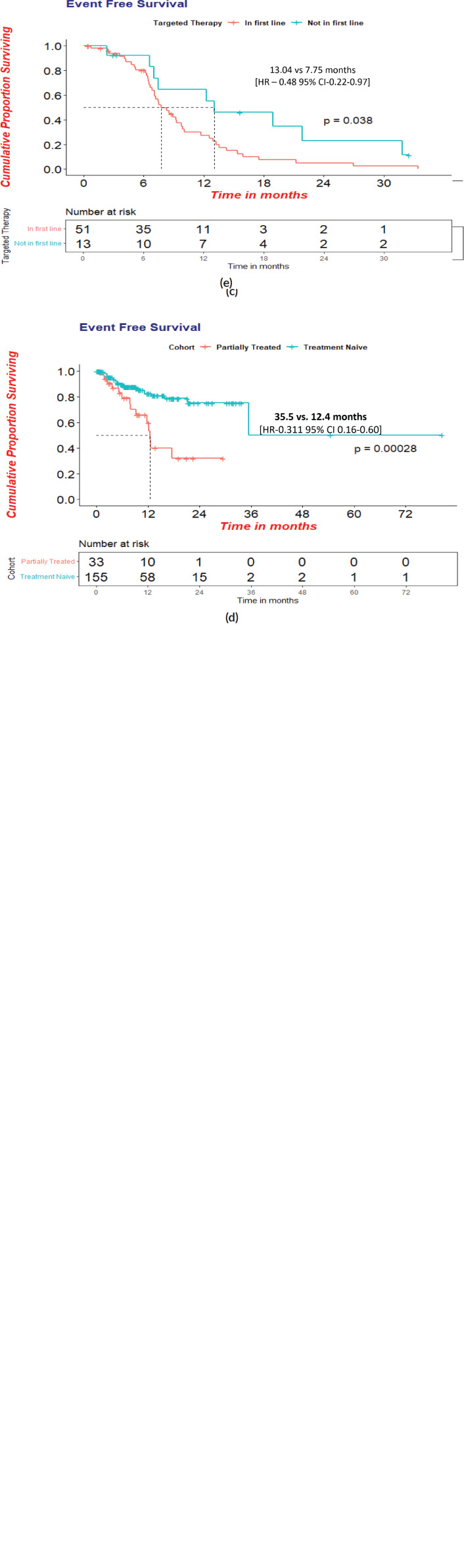
(a): Event free survival in overall cohort(*N* = 188). (b): Event free survival with respect to receptor status. (c): Event free survival in patients with or without brain metastasis at presentation. (d): Event free survival in treatment- naïve at presentation versus prior-treated at peripheral centres patients. (e): Event free survival in HER2+cohort who received targeted therapy in first line.

The median EFS in the treatment-naïve versus prior-treated women was statistically significant with 35.5 (95% CI:12.24–58.72) months versus 12.4 (95%CI:11.45–13.51) [HR−0.525: *p*-0.001] months. The HER2+ women who received targeted therapy in the first line had a significantly superior median EFS of 13.0 versus 7.7 months (HR −0.465:CI 0.22−0.57: *p*-0.038) (Figure 3a–d).

**Figure 4.**
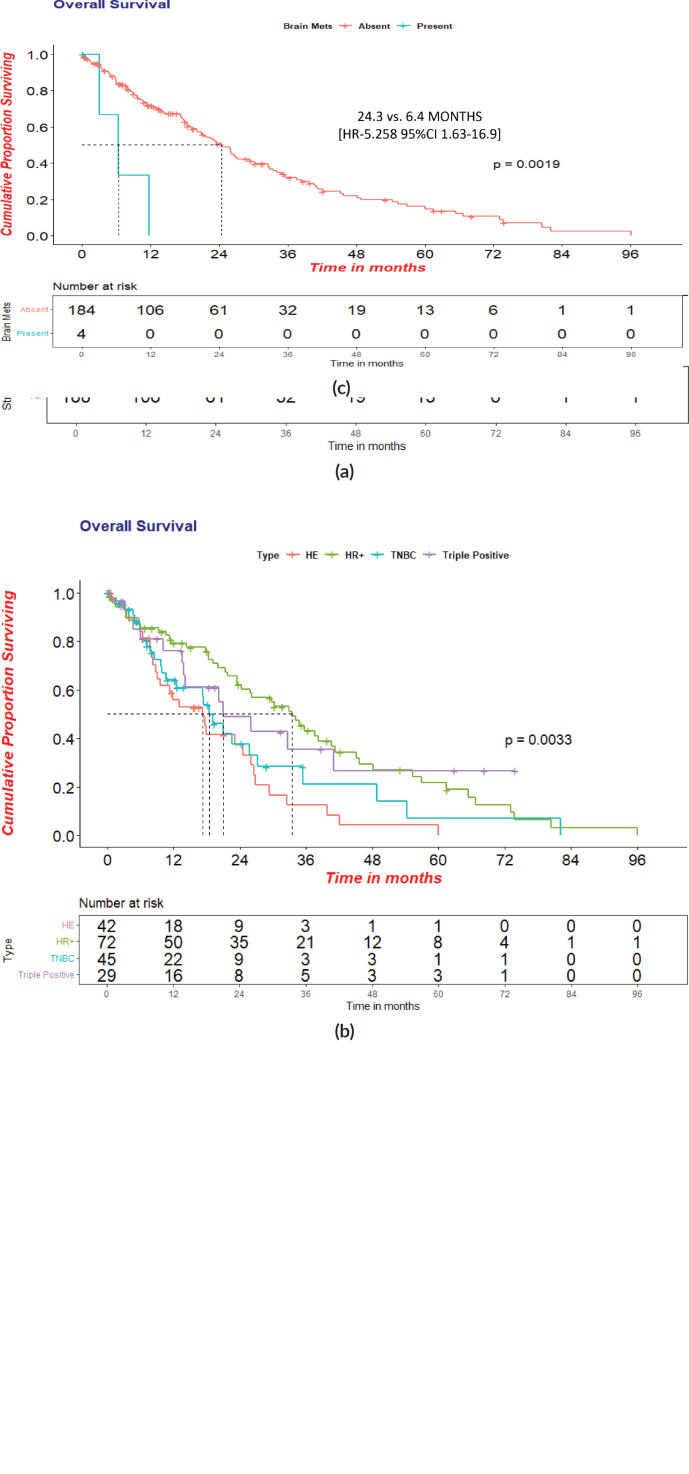
(a): Overall survival in overall cohort(*N* = 188). (b): Overall survival with respect to receptor status. (c): Overall survival in patients with or without brain metastasis at presentation.

The cohort’s median OS was 23.3 (95% CI = 19.5 −27.4) months. Women with HR+ status had a statistically significant OS of 33.5 (95% CI- 24.57–42.45, HR- 0.45, *p*- 0.001) months. The median OS in TNBC, HER2+ and TP was 18.5 months, 17.2 months and 21.0 months, respectively. Further analysis showed a statistically significant OS in women without brain metastasis versus those with at the baseline of 24.3 versus 6.4 months in the overall cohort. [(HR-5.258; 95%CI 1.63−16.9; *p*-0.002)]. There was no statistical significance in OS between the age groups <35 years versus ≥35 years, i.e., 21.8 versus 28.6 months, respectively. Survival statistics with respect to receptor subtypes are represented in Figure 4a–4c and Table 2.

*Toxicities***: The therapies were well tolerated.** Chemotherapy toxicity of grade III/IV were evaluated in 120 women, of which febrile Neutropenia (FN) was seen in 8 (6.6%),7 (5.6%), thrombocytopenia in 5 (4.16%), vomiting in 5 (4.16%) and peripheral neuropathy in 10 (8.3%). There was one toxicity-related death due to FN with septicemia in a TNBC woman on first-line therapy with anthracycline-based combination chemotherapy who had with significant tumour and symptom burden with pulmonary and hepatic visceral crisis.

## Discussion

To the best of our knowledge, this study represents India’s largest, single-centre cohort of women with denovo metastatic young breast cancer. The selection of a biological age cutoff of <40 years for categorising individuals as YBC was based on data from the European School of Oncology and the European Society for Medical Oncology. This age threshold is well-acknowledged in consensus guidelines for breast cancer management in young women, recognising the unique health concerns of this demographic, encompassing aspects such as fertility preservation, genetic considerations and psychosocial well-being [4]. The classification of women aged 35 years or below into the v-YBC subgroup aligns with the studies conducted by Liukkonen *et al* [5] and Fabiano *et al* [6].

Our study had a higher overall dnmYBC proportion, among the total breast cancer, i.e., 16.7 % compared to the documented 1%–7% prevalence in HIC [18, 19–21], however, comparable to other low and middle-income countries [22]. The proportion of YBC was 2.5% of all breast cancers, comparable to the European POSH cohort study [23]. The higher proportion of dnmYBC than the recurrent metastatic disease could be attributed partly to the cohort’s inherent young aggressive disease profile, inclusive of biology and stage at presentation, added by the delayed diagnosis due to logistics and social reasons resulting in upstaging (the median duration of symptoms ~ was about 6 months). Additionally, referral dynamics favouring cases with higher tumour burdens are being referred, while localised disease is often managed in peripheral healthcare facilities, wherein even general surgeons operate breast cancer patients [40]; this also contributed to this observation. The cohort has a higher loco-regional burden as well in the majority (91.4%), which is comparable to the POSH and other studies [24–26,]. Notably, among all age groups, women with lower socioeconomic status, young Black, Hispanic and Native American women, and Asians are more likely to present with advanced disease [27–31]. This again underscores the need to create awareness and feasible screening modalities like clinical breast examination, as shown by the study by Mittra *et al* [32]. Our study showed a predominance of the cumulative HR + subgroup (62.2%), which aligns with the study by an Italian and a Chinese YBC study, although still, the remaining 38% had aggressive biology, which is proportionately higher than in the older population [14, 23, 31].

The patterns of metastasis and their clinical implications in this age group have remained under explored. We identified an elevated risk of visceral metastasis (78%) and liver metastasis (57%), which is similar to the other studies [31, 33, 34]. An analysis involving 14,403 women from the epidemiological strategy and medical economics (ESME) database found that mYBC women were more likely to exhibit visceral metastases than bone metastases [30]. Similarly, a survey of 6,640 women revealed a higher risk of brain and liver metastases [31]. Although Chen *et al* [33] identified that women under 50 were less likely to present with lung metastasis (5.9%) when compared to the older population. Our current study exhibited a notably higher occurrence of lung metastases (38%), perhaps due to higher TNBC and ethnic variations in this cohort. Brain metastases were particularly prominent among individuals with the HER2+ subtype and visceral metastasis in TNBC subtypes, while HR+ women displayed a propensity for nodal and skeletal metastases. These findings parallel a few other studies inclusive of a cohort study involving 2,248 women conducted by Hung *et al* [34] [35–39].

Among the HR + women, 18.0 % received ovarian suppression with Leuprolide and 11 (5.85%) underwent RTOA, which suggests a preference for medical means in advanced disease settings rather than ablative-radical measures, which induce sudden menopause and at times severe symptoms. Forty-two (58.3%) of HR+ women received chemotherapy in the first-line setting, of which 12 (28.5%) women were in visceral crisis, 16 (38.0%) in impending visceral crisis, 9 (21.4%) started treatment from peripheral centres prior to presenting to our Centre wherein there is still predilection use chemotherapy rather than endocrine therapy in young women with resultant over treatment. A study by Frank *et al* [30] also showed higher use of chemotherapy (80.5%) over endocrine therapy (70.1%). Doublet therapy was received by 75 (39.8%), three drug combinations in 18 (9.6%) and single drug in 16 (8.5%), depending upon the need to achieve a fast response. It is important to note that this clinical benefit is modest with aggressive multi-agent chemotherapy and accompanied by an increased risk of toxicity, as outlined in a Cochrane review [40].

HER2+ subgroup necessitates anti HER 2 targeted therapy [41]. However, the accessibility was an issue in this slightly old cohort of mYBC women, wherein only 17% received HER2-targeted therapy in first line and 56.3% in later lines. This can be related to their poor socio-economic background. The patients who received anti-HER2 understandably faired significantly better. Now, with the availability of generic molecules and support provided by government schemes and social services, a large majority of women are able to access targeted therapy like Central Government Health Scheme (Ayushman Bharat – Pradhan Mantri Jan Arogya Yojana, Health Minister’s Discretionary Grant and so on. The first CDK-4/6 inhibitor was available for use in India from October 2016, hence the low rate of use, as cost and accessibility were prohibitive factors.

At a median follow-up of 39.8 months, our study demonstrated an overall survival of 23.3 months, a figure that is comparable to findings from Western studies [13, 30]. Notably, the study did not identify any survival distinctions between women of young and very young age with dnmYBC. The difference has been reported by Bouferraa *et al* in a non-metastatic setting [42]. Notably, the research revealed a significant improvement in survival among women who received upfront HER2-directed therapy among HER2+ women, underscoring the socioeconomic disparities within our population and the limitations in delivering standard-of-care targeted therapy.

The results emphasise the need for urgent national and global initiatives to narrow healthcare disparities and improve the well-being of young women with denovo metastatic breast cancer. Approximately 18% of the cohort started chemotherapy in peripheral settings, leading to poorer outcomes, emphasising the need to increase awareness about early referral. These findings also enhance our insights into mYBC and reveal the intricate factors impacting patient outcomes.

We conclude that denovo mYBC is associated with aggressive features and course. Prognostic factors include tumour characteristics, receptor status, brain metastasis, inadvertent prior treatment and appropriate use of targeted therapy early in the disease course. Awareness, early diagnosis and prompt treatment are warranted to improve patient outcomes. We need to work towards closing this cancer divide and expanding accessibility to replicate the results of newer molecules in the LMICs.

## Conflict of interest

The corresponding author declares no conflicts of interest from any of the authors.

## Funding

None.

## Ethical approval

The study was approved by the Institutional Ethics Committee of Tata Memorial Hospital, Mumbai and was conducted in accordance with the principles of the declaration of Helsinki principles and Good Clinical Practice guidelines. The study is registered with the Clinical Trials Registry of India (CTRI/2021/01/030325).

## Figures and Tables

**Table 1.** Baseline characteristics.

Baseline characteristics	Frequency (*N* = 188)
Age	Median 35.50 (IQR:31–38) years
Sites of metastasis*	
Visceral organs	146 (77.6%)
Liver	108 (57.4%)
Lungs	71 (37.7%)
Brain	4 (2.1%)
Skeletal	
Bone only	17 (9.0%)
Bone + viscera	132 (70.2%)
Non regional nodes	67 (35.6%)
Grade	*N* = 188
II	21 (11.2%)
III	167 (88.8%)
Receptor status	*N* = 188
HR+	72 (38.3%)
HER2+	42 (22.4%)
TNBC	45 (23.9%)
TP	29 (15.4%)

Abbreviations; IQR: Interquartile range, HR+: Hormone Receptor Positive, HER2+: human epidermal growth factor receptor 2, TNBC: Triple Negative Breast Cancer, TP: Triple Positive,

* since some patients had multiple sites of metastasis, the N number is not equal to 188

**Table 2.** Predicted overall survival at 1 year, 2 years, 3 years and 5 years, shown in months with a 95% confidence interval in relation to receptor status.

Year	HER2+ (95%CI)	HR+ (95%CI)	TNBC (95%CI)	TP (95%CI)
1	55.8 (74.7–41.7)	79.3 (89.6–70.1)	64.2 (81.3–50.6)	76.2 (95.0–61.2)
2	37.3 (59.3–23.4)	62.1 (75.5–51.1)	37.7 (60.3–23.6)	48.8 (77.0–30.9)
3	12.4 (34.9–4.4)	43.2 (58.3–32.0)	21.2 (49.0–9.2)	35.6 (67.2–18.8)
5	4.1 (27.8–0.6)	21.6 (37.7–12.3)	7.1 (43–1.2)	26.7 (62.5–11.4)

CI, Confidence interval; HER2+, Human epidermal Growth Factor receptor 2 positive; TNBC, Triple negative breast cancer; HR+, Hormone receptor positive; TP, Triple positive

**Table S1.** Treatment characteristics.

*N* = 188	HR+ *N* = 72	HER2+ *N* = 42	TP *N* = 29	TNBC *N* = 45
First line therapy				
Chemotherapy				
Taxane based	5 (6.9%)	13 (30.9%)	4 (13.7%)	10 (22.2%)
Anthracycline based.	34 (47.3%)	26 (61.9%)	20 (68.9%)	33 (73.3%)
Targeted therapy (in combination/ single agent)	0 (0.00%)	9 (21.4%) (Trastuzumab	4 (13.7%) (Trastuzumab	0 (0.0%)
Others	3 (4.1%)	2 (4.7%)	0 (0.0%)	2 (4.5%)
Hormone therapy	30 (41.7%)	0 (0.0%)	2 (6.9%)	0 (0.0%)
Second line therapy				
Chemotherapy				
Taxane based	20 (37.7%)	12 (50.7%)	4 (23.5%)	13 (56.5%)
Anthracycline based.	5 (9.4%)	1 (4.2%)	2 (11.7%)	8 (34.7%)
Targeted therapy (in combination/ single agent)	2 (2.7%)	13 (54.1%) Trastuzumab-10 Lapatinib-3	6 (35.3%) Trastuzumab-3 Lapatinib-3	0 (0.0%)
Others	4 (3.7%)	5 (20.8%)	1 (5.9%)	2 (8.8%)
Hormone therapy	28 (52.8%)	0 (0.0%)	4 (23.5%)	0 (0.0%)
Thirdline therapy				
Chemotherapy				
Taxane based	6 (26.4%)	4 (35.7%)	2 (37.9%)	1 (47.0%)
Anthracycline based.	5 (7.0%)	1 (14.2%)	6 (20.6%)	4 (6.0%)
Targeted therapy (in combination/single agent)	3 (2.7%) Ribociclib-2 Palbociclib-1	7 (38.1%) Trastuzumab-3 Lapatinib-5	3 (27.5%) Trastuzumab-3 Palbociclib-1	0 (0.0%)
Others	5 (5.5%)	2 (12.0%)	3 (10.3%)	8 (47.0%)
Hormone therapy	14 (58.4%)	0 (0.0%)	3 (51.7%)	0 (0.0%)
Fourth line therapy				
Chemotherapy				
Taxane based	4 (18.1%)	1 (12.5%)	0 (0.0%)	0 (0.0%)
Anthracycline based.	1 (4.5%)	1 (12.5%)	0 (0.0%)	0 (6.0%)
Targeted therapy (in combination/single agent)	3 (13.6%)	3 (37.5%) TDM1-1 Trastuzumab-1 Lapatinib-1	3 (75.0%) Lapatinib-3	0 (0.0%)
Others	5 (22.7%)	3 (37.5%)	0 (0.0%)	6 (100.0%)
Hormone therapy	10 (45.4%)	0 (0.0%)	2 (50.0%)	0 (0.0%)
Fifth line therapy				
Chemotherapy				
Taxane based.	0 (18.1%)	1 (20.0%)	0 (0.0%)	0 (0.0%)
Anthracycline based.	0 (4.5%)	1 (20.0%)	0 (0.0%)	0(6.0%)
Targeted therapy (in combination/single agent)	1 (10.0%)	3 (20.0%) Trastuzumab-1 Lapatinib-2	1 (25.0%) Palbociclib-1	0 (0.0%)
Others	5 (50.0%)	3 (60.0%)	0 (0.0%)	2 (100.0%)
Hormone therapy	5 (50.0%)	0 (0.0%)	3 (75.0%)	0 (0.0%)
Sixthline therapy				
Chemotherapy				
Taxane based.	2 (50.0%)	0 (0.0%)	0 (0.0%)	0 (0.0%)
Anthracycline based.	1 (25.0%)	0 (0.0%)	0 (0.0%)	0 (6.0%)
Targeted therapy (in combination/single agent)	0 (10.0%)	0 (0.0%)	1 (50.0%) Trastuzumab-1	0 (0.0%)
Others	1 (25.0%)	0 (0.0%)	1 (50.0%)	1 (100.0%)
Hormone therapy	0 (0.0%)	0 (0.0%)	0 (0.0%)	0 (0.0%)

HR+- Hormone Receptor Positive, HE- HER2 Enrich, TNBC- Triple Negative Breast CANCER, TP- Triple Positive Others – Gemcitabine-Carboplatin, Capecitabine, Vinorelbine, Eribulin, Oral Metronomic Chemotherapy (etoposide, cyclophosphamide)

## References

[1] Sung H, Ferlay J, Siegel RL (2021). Global cancer statistics 2020: GLOBOCAN estimates of incidence and mortality worldwide for 36 cancers in 185 countries. CA Cancer J Clin.

[2] Mehrotra R, Yadav K (2022). Breast cancer in India: present scenario and the challenges ahead. World J Clin Oncol.

[3] Ferlay JEM, Lam F, Colombet M (2018). Bray F Global Cancer Observatory: Cancer Today.

[4] Paluch-Shimon S, Cardoso F, Partridge AH ESO-ESMO 4th international consensus guidelines for breast cancer in young women (BCY4). Ann Oncol.

[5] Liukkonen S, Leidenius M, Saarto T (2011). Breast cancer in very young women. Eur J Surg Oncol J Eur Soc Surg Oncol Br Assoc Surg Oncol.

[6] Fabiano V, Mando P, Rizzo M (2020). Breast cancer in young women presents with more aggressive pathological characteristics: a retrospective analysis from an Argentine national database. JCO Glob Oncol.

[7] Arumugham R, Raj A, Nagarajan M (2014). 327P—survival analysis of breast cancer patients treated at a tertiary care centre in southern India. Ann Oncol.

[8] Maurya AP, Brahmachari S (2020). Current status of breast cancer management in India. Indian J Surg.

[9] Anders CK, Hsu DS, Broadwater G (2008). Young age at diagnosis correlates with a worse prognosis and defines a subset of breast cancers with shared patterns of gene expression. J Clin Oncol.

[10] Anders CK, Johnson R, Litton J (2009). Breast cancer before age 40 years. Semin Oncol.

[11] Azim HA, Michiels S, Bedard PL (2012). Elucidating the prognosis and biology of breast cancer in young women using gene expression profiling. Clin Cancer Res.

[12] Bharat A, Aft RL, Gao F (2009). Patient and tumor characteristics are associated with increased mortality in young women (≤ 40 years) with breast cancer. J Surg Oncol.

[13] Gobbini E, Ezzalfani M, Dieras V (1990). Time trends of overall survival among metastatic breast cancer patients in the real-life ESME cohort. Eur J Cancer Oxf Engl.

[14] Tagliabue G, Fabiano S, Contiero P (2021). Molecular subtypes, metastatic pattern and patient age in breast cancer: an analysis of Italian Network of Cancer Registries (AIRTUM) data. J Clin Med.

[15] Allison KH, Hammond ME, Dowsett M (2020). Estrogen and progesterone receptor testing in breast cancer: ASCO/CAP guideline update. J Clin Oncol.

[16] Wolff AC, Somerfield MR, Dowsett M (2013). Recommendations for human epidermal growth factor receptor 2 testing in breast cancer: American Society of Clinical Oncology/College of American Pathologists clinical practice guideline update. J Clin Oncol.

[17] Bajpai J, Ventrapati P, Joshi S (2021). Unique challenges and outcomes of young women with breast cancers from a tertiary care cancer centre in India. The Breast.

[18] Daily K, Douglas E, Romitti PA (2021). Epidemiology of de novo metastatic breast cancer. Clin Breast Cancer.

[19] McKenzie HS, Maishman T, Simmonds P (2020). Survival and disease characteristics of de novo versus recurrent metastatic breast cancer in a cohort of young patients. Br J Cancer.

[20] Brandt J, Garne JP, Tengrup I (2015). Age at diagnosis in relation to survival following breast cancer: a cohort study. World J Surg Oncol.

[21] Johnson RH, Chien FL, Bleyer A (2013). Incidence of breast cancer with distant involvement among women in the United States, 1976 to 2009. JAMA.

[22] Soares LR, Freitas-Junior R, Curado MP (2020). Low overall survival in women with de novo metastatic breast cancer: does this reflect tumor biology or a lack of access to health care?. JCO Global Oncol.

[23] Copson E, Eccles B, Maishman T (2013). Prospective observational study of breast cancer treatment outcomes for UK women aged 18–40 years at diagnosis: the POSH study. J Natl Cancer Inst.

[24] Wang Z, Cheng Y, Chen S (2020). Novel prognostic nomograms for female patients with breast cancer and bone metastasis at presentation. Ann Transl Med.

[25] Han Y, Wang J, Sun Y (2020). Prognostic model and nomogram for estimating survival of small breast cancer: a SEER-based analysis. Clin Breast Cancer.

[26] Liu D, Wu J, Lin C (2020). Breast subtypes and prognosis of breast cancer patients with initial bone metastasis: a population-based study. Front Oncol.

[27] Walsh SM, Zabor EC, Flynn J (2020). Breast cancer in young black women. Br J Surg.

[28] Gorin SS, Heck JE, Cheng B (2006). Delays in breast cancer diagnosis and treatment by racial/ethnic group. Arch Intern Med.

[29] Harper S, Lynch J, Meersman SC (2009). Trends in area-socioeconomic and race-ethnic disparities in breast cancer incidence, stage at diagnosis, screening, mortality, and survival among women ages 50 years and over (1987–2005). Cancer Epidemiol Biomarkers Prev.

[30] Frank S, Carton M, Dubot C (2020). Impact of age at diagnosis of metastatic breast cancer on overall survival in the real-life ESME metastatic breast cancer cohort. The Breast.

[31] Zhang W, Wu S, Liu J (2022). Metastasis patterns and prognosis in young breast cancer patients: a SEER database analysis. Front Oncol.

[32] Mittra I, Mishra GA, Dikshit RP (2021). Effect of screening by clinical breast examination on breast cancer incidence and mortality after 20 years: prospective, cluster randomised controlled trial in Mumbai. BMJ.

[33] Chen MT, Sun HF, Zhao Y (2017). Comparison of patterns and prognosis among distant metastatic breast cancer patients by age groups: a seer population-based analysis. Sci Rep.

[34] Hung MH, Liu CY, Shiau CY (2014). Effect of age and biological subtype on the risk and timing of brain metastasis in breast cancer patients. PLoS One.

[35] Lin NU, Bellon JR, Winer EP (2004). CNS metastases in breast cancer. J Clin Oncol.

[36] Heitz F, Harter P, Lueck HJ (2009). Triple-negative and HER2-overexpressing breast cancers exhibit an elevated risk and an earlier occurrence of cerebral metastases. Eur J Cancer.

[37] Heitz F, Rochon J, Harter P (2011). Cerebral metastases in metastatic breast cancer: disease-specific risk factors and survival. Ann Oncol.

[38] Arvold ND, Taghian AG, Niemierko A (2011). Age, breast cancer subtype approximation, and local recurrence after breast-conserving therapy. J Clin Oncol.

[39] Ji L, Cheng L, Zhu X (2021). Risk and prognostic factors of breast cancer with liver metastases. BMC Cancer.

[40] Carrick S, Parker S, Wilcken N (2005). Single agent versus combination chemotherapy for metastatic breast cancer. Cochrane Database Syst Rev.

[41] Dawood S, Broglio K, Buzdar AU (2010). Prognosis of women with metastatic breast cancer by HER2 status and trastuzumab treatment: an institutional-based review. J Clin Oncol.

[42] Bouferraa Y, Haibe Y, Chedid A (2022). The impact of young age (<40 years) on the outcome of a cohort of patients with primary non-metastatic breast cancer: analysis of 10-year survival of a prospective study. BMC Cancer.

[43] Edge SB, Byrd DR, Compton CC (2010). AJCC Cancer Staging Manual 7th edn (New York: Springer).

[44] Eisenhauer EA, Therasse P, Bogaerts J (2009). New response evaluation criteria in solid tumours: revised RECIST guideline (version 1.1). Eur J Cancer.

[45] National Cancer Institute (2006). Common Terminology Criteria for Adverse Events (CTCAE) v3.0 [Internet].

